# Paramagnetic gold in a highly disordered Au-Ni-O alloy

**DOI:** 10.1038/s41598-019-49457-7

**Published:** 2019-09-11

**Authors:** A. Stamatelatos, P. Poulopoulos, A. Goschew, P. Fumagalli, E. Sarigiannidou, L. Rapenne, C. Opagiste, S. Grammatikopoulos, F. Wilhelm, A. Rogalev

**Affiliations:** 1 Materials Science Department, University of Patras, 26504 Patras, Greece; 2 Institut für Experimentalphysik, Freie Universität Berlin, Arnimallee 14, D-14195 Berlin-Dahlem, Germany; 3 Univ. Grenoble Alpes, CNRS, Grenoble INP, LMGP, 38000 Grenoble, France; 4 Institut Néel, CNRS, UGA, Grenoble INP, F-38000 Grenoble, France; 5 Department of Mechanical Engineering, Technological Educational Institute (T.E.I.) of Western Greece, M. Alexandrou 1, Patras, Greece; 6 European Synchrotron Radiation Facility (ESRF), B.P.220, 38043 Grenoble, France

**Keywords:** Magnetic properties and materials, Surfaces, interfaces and thin films

## Abstract

Magnetic materials are usually classified into a distinct category such as diamagnets, paramagnets or ferromagnets. The enormous progress in materials science allows one nowadays, however, to change the magnetic nature of an element in a material. Gold, in bulk form, is traditionally a diamagnet. But in a ferromagnetic environment, it can adopt an induced ferromagnetic moment. Moreover, the growth of gold under certain conditions may lead to a spontaneous ferromagnetic or paramagnetic response. Here, we report on paramagnetic gold in a highly disordered Au–Ni–O alloy and focus on the unusual magnetic response. Such materials are mainly considered for plasmonic applications. Thin films containing Au, Ni and NiO are fabricated by co-deposition of Ni and Au in a medium vacuum of 2 × 10^−2^ mbar. As a result, Au is in a fully disordered state forming in some cases isolated nanocrystallites of up to 4 nm in diameter as revealed by high resolution transmission electron microscopy. The disorder and the environment, which is rich in oxygen, lead to remarkable magnetic properties of Au: an induced ferromagnetic and a paramagnetic state. This can be proven by measuring the x-ray magnetic circular dichroism. Our experiments show a way to establish and monitor Au paramagnetism in alloys.

## Introduction

Gold (Au) is an inert noble transition metal with 5*d* electrons. The crystal structure is face-centered cubic (*fcc*). It is known as a typical diamagnetic material with negative magnetic susceptibility. As an isolated atom, Au has completely filled 5*d* states, so there is no magnetic moment. In metallic form, there is a small number of holes in the 5*d* band due to self-hybridization effects. During the last 15 years, outstanding scientific research on Au has been performed using element-specific techniques carried out in synchrotron-radiation facilities. It has been demonstrated by x-ray magnetic circular dichroism (XMCD) that Au can acquire an induced magnetic moment when it forms alloys or layered-film structures with 3*d* ferromagnetic transition metals^1–3^. Ferromagnetism in tiny Au nanoparticles, without or with thiol-group capping, has also been reported^4–6^. More recently, paramagnetism in both, bulk Au and Au nanoparticles has attracted the scientific interest: Suzuki *et al*. probed Pauli and orbital paramagnetic state in bulk gold by XMCD^7^. Bartolomé *et al*. showed that for Au nanoparticles deposited on a Sulfolobus acidocaldarius S layer, the magnetic moment per paramagnetic Au atom was 25 times larger than in other Au nanoparticles measured at that time^8^. From these studies, an additional interest emerged for further investigation of Au paramagnetism on different probe systems and in various environments.

According to ref.^8^, the key factor for discovering paramagnetic gold is the increase in the number of 5*d* holes. This may originate from a charge transfer from Au atoms in nanoparticles to other atoms and/or the existence of Au in the form of tiny crystals or clusters with open bonds. Therefore, in this work, we intentionally fabricated a highly disordered system containing tiny nanoparticles of Au and Ni. A large percentage of Ni is oxidized. By means of XMCD experiments, we record for Au an induced ferromagnetic state for small magnetic fields followed by a paramagnetic state up to 17 T. The two contributions can be separated. Our experiments show a way to establish and probe Au paramagnetism in alloys. Up to now, only one paper reported on Au paramagnetism, exhibited by nanoclusters grown on an archaeal-cell-wall surface layer^8^. Our work shows a more systematic way to grow nano-systems in which it is possible to detect Au paramagnetism. Last but not least, Au in NiO is an interesting, plasmonic system^9–11^. It finds many applications, amongst others, in catalysis, photovoltaics and gas sensing.

## Results

**Figure 1:**
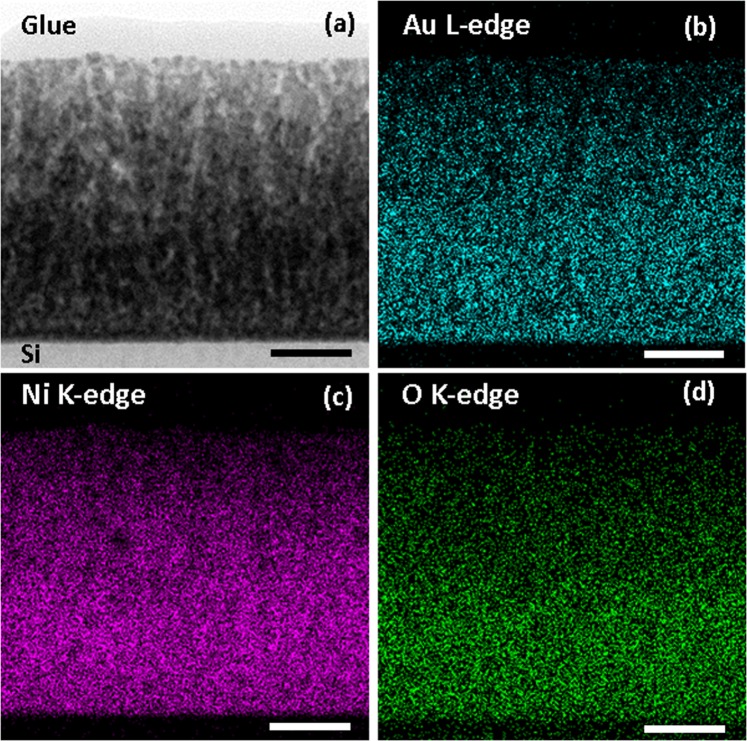
(**a**) Annular bright-field STEM image of Au-Ni-O thin film, (**b–d**) corresponding EDS-STEM elemental mapping of the gold, nickel and oxygen elements, respectively. The scale bar is 30 nm.

In Fig. 1, a cross-section scanning transmission electron microscopy (STEM) image and the results of energy-dispersive X-ray spectroscopy (EDS) analysis are shown. The chemical analysis presented by EDS-STEM images indicates that Au atoms are homogeneously distributed in the Au-Ni-O thin film. Within the accuracy of the EDS detector of 5%, the film contains about 8 at. % Au, with about two thirds of Ni being oxidized.

**Figure 2:**
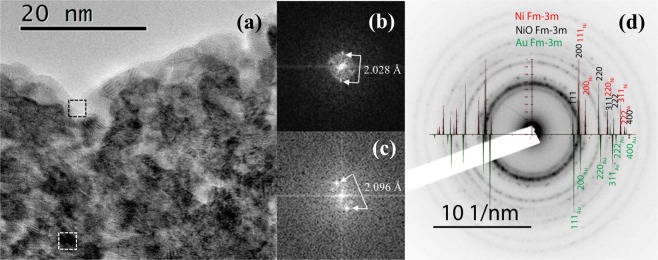
(**a**) HRTEM image of Au-Ni-O thin film close to the surface of the 100 nm film, (**b**,**c**) FFT of the square areas noted in (**a**). Interplanar distances extracted from black areas correspond to Au and from light-color areas to NiO. (**d**) Electron-diffraction pattern of the 100 nm Au-Ni-O film.

In Fig. 2(a), a cross-section high-resolution transmission electron microscopy (HRTEM) image is shown. We clearly see the polycrystalline nature of the Au-Ni-O thin film. The average size of the crystallites is less than 5 nm in diameter. They feature various shapes. The overall thickness of the film, determined with high precision, was 103 ± 1 nm. Electron diffraction [Fig. 2(d)] confirms the presence of both, NiO and Ni Fm-3m phases. The presence of Au rings could not be verified in the electron diffraction of large areas of the image due to fact that the Au interplanar distances are very close to the NiO ones. Furthermore, taking into account the enlargement of the NiO rings due to the small size of the crystallites, they most probably overlap with the ones from Au.

Since we could not separate NiO from Au contributions by electron diffraction, a thin TEM lamella has been prepared. To enhance the contrast, a series of under-focus HRTEM images close to the thinnest area of the TEM lamella has been taken in order to measure directly the interplanar distances. From fast Fourier transform (FFT), extracted from small black and small bright areas in Fig. 2(a), the measured interplanar distances were found to correspond to Au [Fig. 2(b)] and to NiO [Fig. 2(c)], respectively. For the majority of grey areas, the size of the Au crystals is smaller than the thickness of the TEM lamella and, as a result, the measured distance is averaged between Au and NiO. Note also that HRTEM in a similar but only 10 nm thick film showed the presence of partially amorphous material; only part of Au was in crystalline form with a few isolated grains up to 2 nm in diameter^11^. In the present work, we could not identify amorphous material, so Au is in cubic *fcc* crystalline form consisting of tiny nanocrystals (smaller than about 4 nm, see Fig. 2(a), black areas). The existence of open bonds (Au in form of incomplete crystals or clusters) cannot be ruled out either. In ref.^11^, a part of Ni was in the hexagonal *hcp* phase. Hexagonal Ni in bulk form does not affect the magnetic properties of its environment as it is non-magnetic^12,13^. In the form of small nanoparticles, *hcp* Ni could also show some antiferromagnetism^14^. However, in the thick Au-Ni-O film of this study no *hcp* Ni was evidenced. The common conclusion of both studies, this one and ref.^11^, is that they show evidence of a highly disordered system.

**Figure 3:**
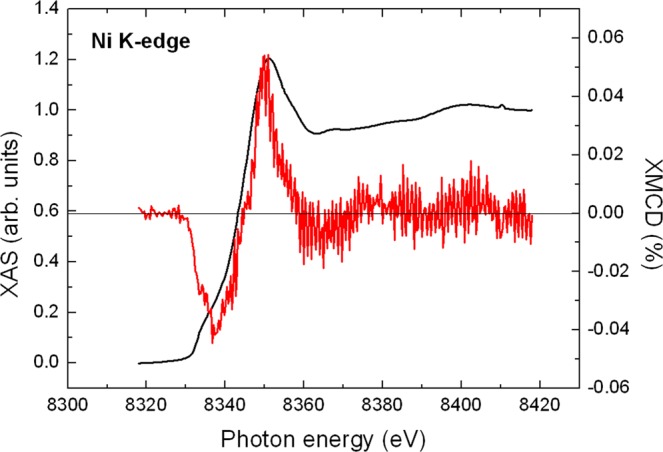
XAS (smooth line) and XMCD (noisy line) recorded at the K edge of Ni in a disordered Au-Ni-O film. The XAS spectrum is normalized to unity.

In Fig. 3, the spectra of X-ray absorption spectroscopy (XAS) and XMCD at the Ni *K*-edge of our film are plotted. The XAS recorded on the film is a linear combination of XAS of Ni in NiO and pure Ni. According to EDS, the total amount of Ni in the sample is 55%, of which 35% is NiO and 20% is Ni metal. The XAS measures all Ni atoms of the sample, i.e. the spectral shape of the XAS is a mixture between NiO and Ni metal. The XAS spectrum resembles more the one of NiO (crystallized in a slightly rhombohedrally-distorted cubic NaCl structure)^15^ than the one of bulk *fcc* Ni^16,17^. This observation confirms the results of EDS and HRTEM that most of Ni is oxidized. On the other hand, the spectral shape of the XMCD signal corresponds for the most part to the one of Ni metal. However, this XMCD signal is much smaller than the one expected for *fcc* Ni. This can be clearly seen by a direct comparison of the XMCD Ni signal of Fig. 3 to the one appearing in ref.^17^ for pure *fcc* bulk Ni. Since NiO is an antiferromagnet, it has zero contribution to the XMCD signal. Hence, the small Ni *K*-edge XMCD is due to the small percentage of *fcc* Ni in the sample. From its amplitude, we can deduce that at most 20% of the Ni atoms belong to ferromagnetic Ni metal. Differences in the line shape of the XMCD as compared to bulk Ni^17^ may arise from the small size effects (low dimensionality) of the Ni *fcc* clusters. These effects may modify the density of states and, consequently, the orbital magnetism, which is probed through the *K*-edge XMCD signal. A further reduction of the signal may originate from disorder. Indeed, disorder was shown to reduce Ni *K*-edge XMCD in NiMnGa alloys^18^. Finally, one has to consider that in some cases disorder and strain may induce a ferromagnetic phase in NiO, see e.g. ref.^19^ and references therein. In this case, the average magnetization increases with decreasing particle size, which is correlated to an increase in the number of uncompensated spins at the surface with respect to the core. Such a phase could also contribute to the Ni XMCD signal.

**Figure 4:**
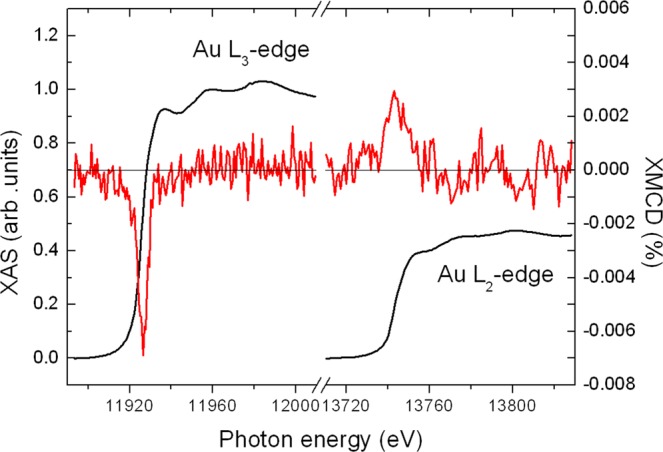
XAS (smooth line) and XMCD (noisy line) recorded at the *L*_3_ and *L*_2_ edge of Au in a disordered Au-Ni-O film. At the *L*_3_ edge, the XAS spectrum is normalized to unity.

In Fig. 4, the XAS and XMCD spectra recorded at 295 K under a field of 17 T at the *L*_3_ and *L*_2_ edge of Au are plotted for the Au-Ni-O film. For the XAS spectra, the ratio of the *L*_3_ and *L*_2_ intensity was normalized to 2.24:1 according to ref.^20^. This value is also very close to the value of 2.2:1 reported by Tyson *et al*.^21^. The finite XMCD signal reveals that Au possesses a magnetic moment. Knowing the direction of the magnetic field and the helicity of the beam, we conclude that Au is polarized parallel to the magnetic field and to the Ni magnetic moment.

**Figure 5:**
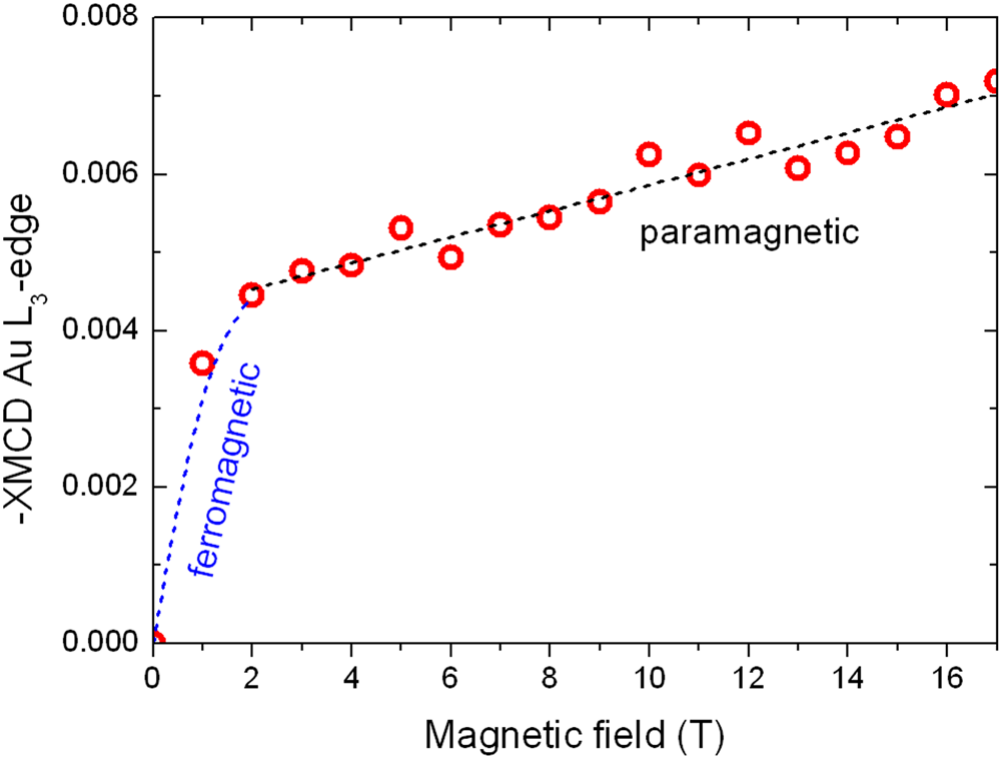
Magnetization curve recorded at the *L*_3_ edge of Au in a disordered Au-Ni-O film. The curve shows (below 1 T) an induced ferromagnetic and (above 1 T) a paramagnetic behavior.

**Figure 6:**
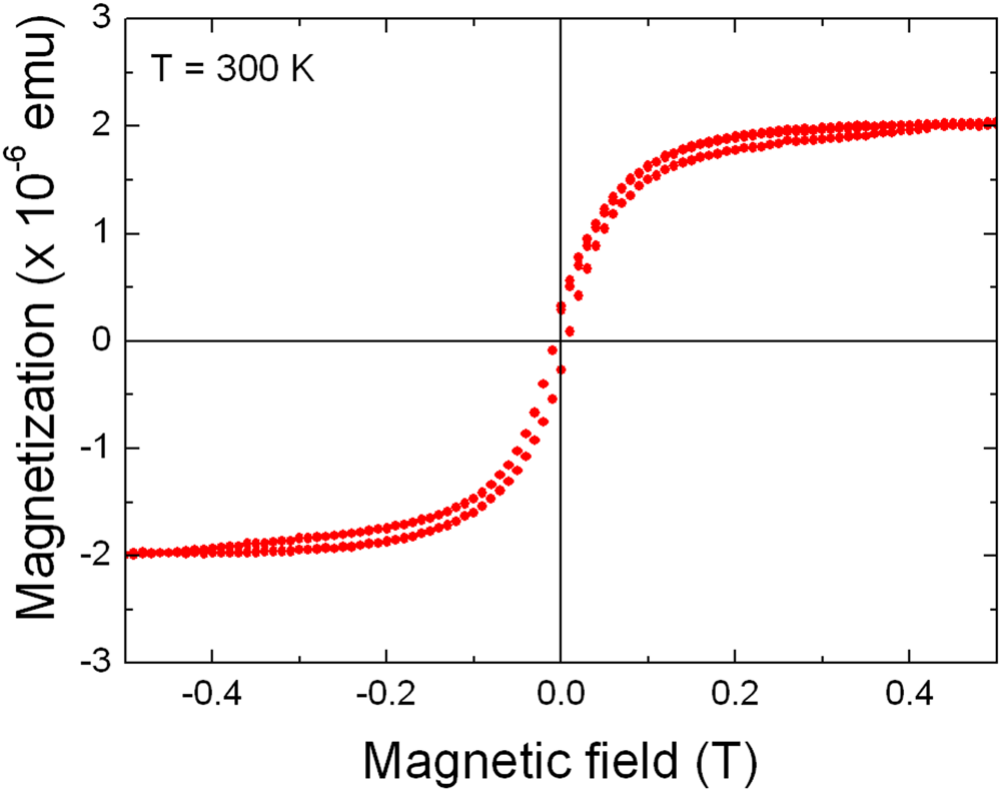
SQUID VSM hysteresis loop recorded with the magnetic field applied parallel to the film surface.

By setting the monochromator at the maximum of the *L*_3_ XMCD signal of Au and increasing the magnetic field to 17 T, we have recorded an element-specific magnetization curve for Au. It is a well-known advantage of the XMCD technique to allow recording of element-specific magnetization curves^22,23^. The result is plotted in Fig. 5. A rather abrupt increase is seen at applied fields lower than 1 T, followed by a slow linear response up to 17 T. Since the field increment is 1 T, the behavior near zero is not known in detail. Nevertheless, the low-field behavior may be attributed to an induced magnetic moment of Au that is spin-polarized by ferromagnetic Ni, Alternatively, it might originate from a polarization of Au by superparamagnetic Ni. To clarify this, magnetization measurements were performed with a Quantum Design superconducting quantum-interference device vibrating-sample magnetometer (SQUID VSM). As illustrated in Fig. 6, a hysteresis loop of the 200 nm thick sample has been recorded at 300 K. The corrected magnetization loop, i.e., after subtraction of a contribution of the diamagnetic Si substrate, is typical for a soft ferromagnetic polycrystalline material with a coercivity of 7.5 mT. The hysteresis is completed at about 0.5 T and the magnetization reaches a saturation value of 2 × 10^−6^ emu.

## Discussion

The induced magnetic moments of Pd, Pt, and even Au, in a ferromagnetic environment, probed by XMCD, was the target of a few papers in the nineties and afterwards. For the first two elements, there were results for magnetic polarization by Fe, Co and Ni^24,25^. Au, as far as we know, is known to be polarized only by Co^1^ or Fe^3^. Therefore, it is reasonable to expect that Ni could also spin-polarize Au, e.g. in ferromagnetic Ni/Au multilayers or NiAu alloys. This is the behavior of Au in our sample under fields up to approximately 1 T, as one can conclude from Figs 5 and 6. Note that NiO is an antiferromagnet and such transition-metal oxides were, up to now, not shown to be able to produce proximity effects even to easily spin-polarizable elements such as Pt^26,27^.

Even more interesting is the observation of the slow linear increase, which is typical for paramagnetic materials. Au is not a paramagnet but it has been shown to possess a very small Pauli paramagnetic susceptibility in bulk^7^. Under special circumstances, in the form of nanoparticles grown on special substrates, it can show a strong paramagnetic behavior^8^. In order to quantify the paramagnetic behavior of Au in our system, the susceptibility of Au has to be determined, which is defined as χ = d*M*/d*H*, where *M* is the magnetization and *H* the magnetic-field strength. Therefore, we applied on the spectra of Fig. 4 the analysis procedure for 4*d* and 5*d* elements with small white-line intensities, developed for Pd and Ag in Fe/Pd multilayers^24^. The XAS spectra at the *L* edges of the transition metal in the film has to be compared with bulk Ag or Au material (usually a pure metallic gold foil is measured as a reference) and the number *n*_h_ of *d* holes of Ag or Au has to be taken into account^24,25^. This method works well if the sample has the *fcc* structure of the reference.

**Figure 7:**
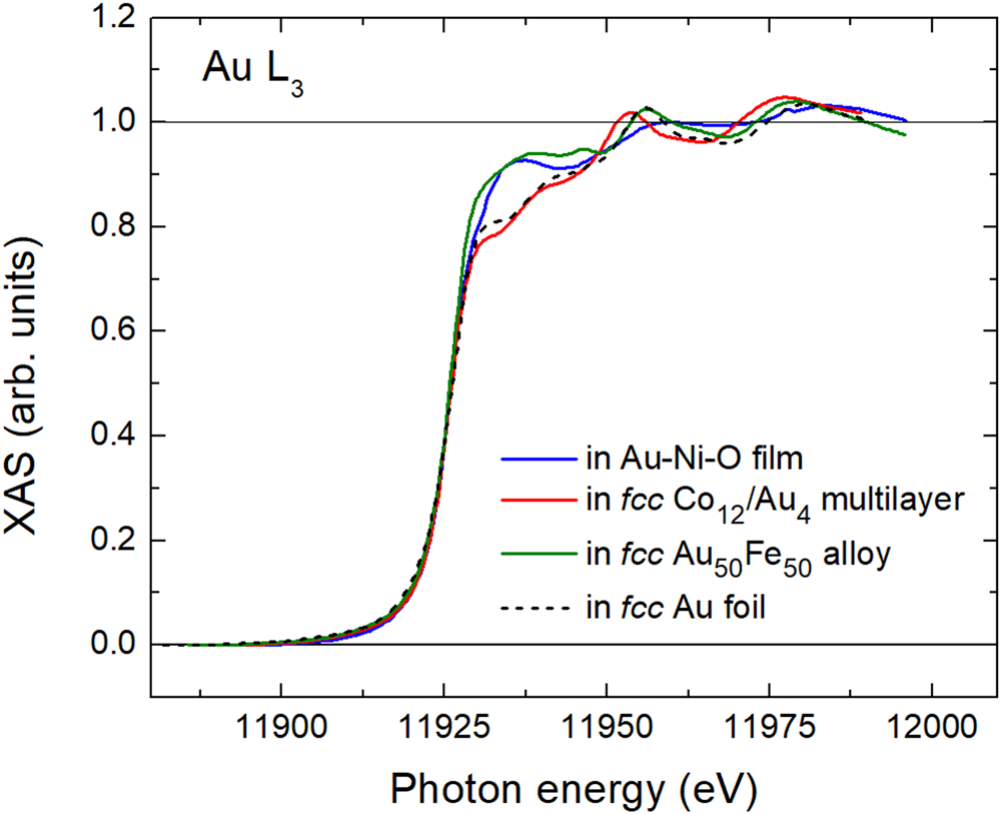
Normalized XAS spectra recorded at the *L*_3_ edge of Au in various specimens as indicated.

In Fig. 7, we plot the XAS spectra at the *L*_3_ edge of Au in our film, in a *fcc* Co_12_/Au_4_ multilayer^1^ (the indices 12 and 4 refer to the number of atomic layers in a multilayer period), in a *fcc* Au_50_Fe_50_ alloy^3^ and in a *fcc* reference gold foil. The reference-foil measurements have been done using a 0.1 mm thick gold foil from Goodfellow with 99.999 at.% purity, which gave the same results as a Au single crystal (99.999 at.% purity, from MaTeck). The energy axis of the spectrum of Au in the reference foil was stretched in order to match the one of Au in the alloy, where the atomic distances are different from bulk Au^23^. One may observe that (i) the spectroscopic features of Au after the edge are quite similar for the latter three *fcc* samples but less for Au in Au-Ni-O. The simple, almost sinusoidal spectroscopic profile of Au in our Au-Ni-O sample reminds of the one of elements in amorphous environments^28^. In our film, we suggest that this is due to large disorder. (ii) In the case of Co/Au multilayers, there is practically no change of the white-line intensity of Au as compared to the reference Au foil. However, white-line intensity increases for both, Au in the AuFe alloy and Au in our sample. In AuFe alloys, this effect was attributed to hybridization between Au 5*d* and Fe 3*d* states, which resulted in a charge transfer from Au to Fe^3^. Obviously, between the less-reactive Co and Au no such charge transfer occurs, leaving the white-line intensity of Au unaltered. One should also expect that the presence of the almost inert Ni would have a similar negligible influence on the white-line intensity of Au in the Au-Ni-O film. Therefore, the white-line intensity increase could be attributed to a probable charge transfer from Au to oxygen or NiO. Indeed, XAS experiments in Au_2_O_3_ revealed a very large increase in the white line at the *L*_3_ absorption edge of Au^29^. On the other hand, a less significant white-line intensity increase, more or less similar to our case, was reported for Au nanoparticles about 10 nm in diameter embedded in a NiO matrix with incorporated hydrogen^15^. Combination of XAS with measurements of the electrochromic effect revealed a charge transfer between NiO and Au^15^. Besides this charge transfer, the size of Au clusters, e.g. in a disordered material, can also be thought responsible for white-line intensity changes^30^. Therefore, both effects could contribute to the white-line increase observed in our film.

The fact that the XAS of Au in our sample does not match the one of *fcc* Au, because the lattice symmetry is different, does not allow analyzing our spectra following ref.^26^. In such a case, an approximate method can be followed, adopted for *hcp* CoCrPt alloys^31^. It has also been used in the evaluation of the Au moment in a *bcc* Au_97_Fe_3_ alloy^3^. After normalizing the XAS spectra, we compare the integrated XMCD spectra of Au in our film to the ones of Au in the pure metallic *fcc* gold foil. This idea is justified by the fundamental principle that the sum rules establish a simple linear relationship between the magnitude of the XMCD signal and the magnetic moment, for a detailed discussion, see ref.^32^. Application of this principle to the spectra of Au in Fig. 4 allows one to estimate with an accuracy of the order of 20% a very small total 5*d* magnetic moment of about 0.016 μ_B_/atom for Au in our film in a field of 17 T. This value is more than one order of magnitude smaller than the 5*d* moment of Au in a Au_50_Fe_50_ alloy. As Fig. 5 shows, practically 60% of this value is due to induced ferromagnetic moment and the rest is a paramagnetic contribution.

By scaling the *y* axis of Fig. 5 in μ_B_/at of Au, one may estimate from the slope of the linear part of the curve the paramagnetic susceptibility of Au. The value we calculate is χ = 3.5 × 10^−7^ μ_B_/at-Oe. In SI units, this amounts to χ ~ 2 × 10^−3^. This value falls well within the range of susceptibility values observed in paramagnetic materials^33^. The value is comparable to the susceptibility at 10 K of Au nanoparticles deposited on an archaeal-cell-wall surface layer^8^. The charge transfer in that case was found to be 0.152 electrons^8^. In our work, we can give only a rough estimate because the white-line intensity in our sample is comparable to the one in a AuFe alloy (Fig. 7). Thus, the charge transfer is about 0.045 electrons. This value may be determined from the Au spectra of ref.^3^ and the use of a Au spectra for an Au_4_Mn alloy, in a similar manner as described in ref.^8^.

An interesting point to be discussed is the fundamental origin of the paramagnetic Au behavior. The most straightforward justification would be the existence of localized moments on the tiny Au nanocrystallites and clusters. In principle, Pauli paramagnetism could have been expected by Au crystallites that are substantially larger than ours, in order that proper electronic bands of Au can develop. Pauli paramagnetism would probably result in smaller susceptibility values^6^ than those determined in this work. However, a few papers suggest that the metallic character is preserved even for small clusters of gold^34^, sodium^35^ and palladium^36^. In the case of Pd, a large susceptibility was observed at low temperatures, while Pauli paramagnetism was noticeable even at room temperature. Therefore, in the present work, our experiments cannot clearly exclude the existence of Pauli paramagnetism.

## Conclusions

In this work, strongly disordered films of Au-Ni-O have been grown. The Au concentration was about 8 at.%. Via XMCD measurements, we observed an induced magnetic moment of Au, showing as a function of applied magnetic field an initially abrupt increase, followed by a slow increase up to 17 T revealing a paramagnetic contribution. The total magnetic moment of Au at 17 T is 0.016 ± 0.003 μ_B_/atom; 60% of it is due to a ferromagnetic contribution and the rest is attributed to Au paramagnetism most probably due to localized moments. A paramagnetic susceptibility χ ~ 2 × 10^−3^ (in SI units) is found for Au; this value falls well within the range of susceptibility values observed in paramagnetic materials. Finally, we have demonstrated that it may not be extraordinary to observe paramagnetic gold in nature. The key idea is to put Au atoms in a disordered state in tiny isolated crystallites (with diameter smaller than about 4 nm as the present works shows) or in clusters surrounded by atoms that may attract some charge from Au, such as oxygen atoms. Our work could open the road for the preparation of samples with similar methodologies in order to make further investigations on the interesting and up to now rare phenomenon of Au paramagnetism.

## Methods

Two films, 100 nm and 200 nm thick, were prepared under identical experimental conditions in medium vacuum with base pressure 2 × 10^−2^ mbar by co-deposition of Au and Ni using direct-current magnetron sputtering. The medium vacuum results in the formation of a precursor-material thin film where a significant amount of oxygen is trapped inside the film. The presence of oxygen and the relatively low deposition temperature, which is room temperature, result in a quenching of the deposited material prohibiting crystal growth. This is obvious for room-temperature growth due to low diffusion rates. The presence of oxygen also results in a decreased size of crystallites. As for most metals during thin-film growth, oxygen is first incorporated in the grain boundaries prohibiting crystal growth, see e.g. ref.^37^. Therefore, as is shown by HRTEM, the thin film consists of tiny nanocrystals while part of it is amorphous. This material was previously shown to produce after annealing high-quality plasmonic thin films^11^.

Annular bright field imaging and EDS were collected by STEM with a JEOL 2100 F FEG microscope operating at 200 kV with 0.2 nm resolution in scanning mode. EDS experiments were carried out with the novel Centurio silicon drift detector with a large solid angle of up to 0.98 steradians from JEOL. HRTEM experiments have been performed in a JEOL 2011 microscope with a point resolution of 0.19 nm. All TEM lamella have been prepared using Multiprep semi-automatic tripode polishing and finished with Ar-ion thinning.

The XMCD experiments were performed at the ID12 beamline of the European Synchrotron Radiation Facility in Grenoble (France) at the *L*_3_ and *L*_2_ edge of Au at 295 K and the *K* edge of Ni at 2 K using highly efficient fluorescence-yield detection mode in backscattering geometry^38^. XAS spectra were recorded under external fields up to 17 T. The XAS spectra at the Ni *K* edge and Au *L*_3_ edge were normalized to unity. The experiments were performed at grazing incidence (15° with respect to the film plane). The progress in third-generation synchrotron-radiation facilities has made possible the detection of the Au XMCD signal at the Au *L* edges with relatively good signal-to-noise ratio for a 100 nm thin film containing only about 8 at. % Au. To exclude any experimental artifacts, the XMCD spectra were recorded either by changing the helicity of the incoming light or by inverting the direction of the external magnetic field.

Finally, magnetic measurements were realized on a 3.5 × 4 mm^2^ piece of a 200 nm thick film with the external magnetic field applied parallel to the film surface at 300 K. These measurements are based on the extraction technique, under magnetic fields up to 5 Tesla, using a SQUID VSM. The diamagnetic signal originating from the silicon substrate was subtracted from the raw measurements.
